# Bacteria and Archaea Synergistically Convert Glycine Betaine to Biogenic Methane in the Formosa Cold Seep of the South China Sea

**DOI:** 10.1128/mSystems.00703-21

**Published:** 2021-09-07

**Authors:** Lingyan Li, Wenting Zhang, Shengjie Zhang, Lei Song, Qinglei Sun, Huan Zhang, Hua Xiang, Xiuzhu Dong

**Affiliations:** a State Key Laboratory of Microbial Resources, Institute of Microbiology, Chinese Academy of Sciences, Beijing, China; b University of Chinese Academy of Sciences, Beijing, China; c China General Microorganism Culture Collection Center, Institute of Microbiology, Chinese Academy of Sciences, Beijing, China; d Institute of Oceanology, Chinese Academy of Sciences, Qingdao, China; ExxonMobil Research and Engineering

**Keywords:** cold seep, GBT reduction and demethylation, bacteria, biogenic methane, cryoprotectant, dimethylglycine, glycine betaine, methanogenic archaea, methanogenic precursor, synergism

## Abstract

Cold seeps are globally widespread seafloor ecosystems that feature abundant methane production and flourishing chemotrophic benthic communities. Chemical evidence indicates that cold seep methane is largely biogenic; however, the primary methane-producing organisms and associated pathways involved in methanogenesis remain elusive. This work detected methane production when glycine betaine (GBT) or trimethylamine (TMA) was added to the sediment microcosms of the Formosa cold seep, South China Sea. The methane production was suppressed by antibiotic inhibition of bacteria, while GBT was accumulated. This suggests that the widely used osmoprotectant GBT could be converted to cold seep biogenic methane via the synergistic activity of bacteria and methanogenic archaea because archaea are not sensitive to antibiotics and no bacteria are known to produce ample methane (mM). 16S rRNA gene diversity analyses revealed that the predominant bacterial and archaeal genera in the GBT-amended methanogenic microcosms included *Oceanirhabdus* and *Methanococcoides*. Moreover, metagenomic analyses detected the presence of *grdH* and *mtgB* genes that are involved in GBT reduction and demethylation, respectively. Two novel species were obtained, including bacterium Oceanirhabdus seepicola, which reduces GBT to TMA, and a methanogenic archaeon, Methanococcoides seepicolus, which produces methane from TMA and GBT. The two strains reconstituted coculture efficiently converted GBT to methane at 18°C; however, at 4°C addition of dimethylglycine (DMG), the GBT demethylation product, was necessary. Therefore, this work demonstrated that GBT is the precursor not only of the biogenic methane but also of the cryoprotectant DMG to the microorganisms at the Formosa cold seep.

**IMPORTANCE** Numerous cold seeps have been found in global continental margins where methane is enriched in pore waters that are forced upward from sediments. Therefore, high concerns have been focused on the methane-producing organisms and the metabolic pathways in these environments because methane is a potent greenhouse gas. In this study, GBT was identified as the main precursor for methane in the Formosa cold seep of the South China Sea. Further, synergism of bacteria and methanogenic archaea was identified in GBT conversion to methane via the GBT reduction pathway, while methanogen-mediated GBT demethylation to methane was also observed. In addition, GBT-demethylated product dimethyl glycine acted as a cryoprotectant that promoted the cold seep microorganisms at cold temperatures. GBT is an osmoprotectant that is widely used by marine organisms, and therefore, the GBT-derived methanogenic pathway reported here could be widely distributed among global cold seep environments.

## INTRODUCTION

Cold seeps are localized areas within cold marine systems that exhibit considerable methane release to seafloor fluids and are therefore termed methane seeps. Methane seeps are enriched in methane-metabolizing chemoautotrophic bacteria and archaea that can transform the carbon and chemical energy stored in methane to biomass and biological energy, respectively, in turn supporting flourishing faunal assemblages (1–4). In addition, cold seeps represent large methane sources that can contribute to global warming. Consequently, the discovery and investigation of cold seeps 40 years ago have led to a focus on the methane-metabolizing organisms within these systems, including the anaerobic methane-oxidizing archaea (5–8).

The stable carbon isotope signatures (δ^13^C) of the cold seep methane suggest biogenic sourcing in some regions, as observed for the methane released from the Sonora cold seep in the Guaymas Basin that exhibits δ^13^C values of about −63%, indicating a presumably dominant proportion of biogenic mixed with abiotic methane (e.g., thermogenic methane) (9–11). A cold seep at Formosa Ridge (also termed site F) is located on the northeastern corner of the continental slope of the South China Sea (SCS) (12), where rich communities of benthic invertebrates are found, indicating that this is an active cold seep. The stable carbon isotope signatures (δ^13^C) of Formosa cold seep Bathymodiolus platifrons tissues containing methanotrophic symbionts are −70.3% on average (13). Moreover, methane of the Haima cold seep in SCS also exhibits δ^13^C values of −72 to −99%. These observations also implicate the presence of biogenic methane in the SCS cold seeps.

Methylotrophic methanogenesis and, specifically, methane production from trimethylamine (TMA) has been conventionally viewed as the primary pathway of ocean ecosystem methanogenesis. Importantly, sulfate-reducing bacteria (SRB) and other bacteria, except for some acetogens (14, 15), in anoxic marine sediments are incapable of metabolizing TMA (16), leaving TMA metabolism solely to methanogens without competition. Quaternary amines, glycine betaine (GBT), and choline are ubiquitous osmolytes used by marine microorganisms and benthic invertebrates and are all precursors of TMA. These compounds are assumed to contribute as metabolites to as much as 90% of total methane production in intertidal coastal sediments (17). In addition, other methyl compounds like methylphosphonate could be used by *Vibrio* species to produce methane. Methane production was also found in oxic seawater of the western North Pacific and its marginal seas (18). TMA-utilizing methanogens in marine ecosystems primarily belong to the genera *Methanococcoides* and *Methanosarcina* (19, 20). However, only a few methanogenic species have been reported to directly use quaternary amines to produce methane, including *Methanococcoides* sp. strain Q3C and *Methanolobus* sp. strain B1d (21). In addition, other *Methanococcoides* strains isolated from marine sediments have been shown to produce methane from choline, dimethylethanolamine (DMEA), and GBT (22, 23). A nonpyrrolysine trimethylamine/corrinoid methyltransferase (MttB) homolog in Desulfitobacterium hafniense (that is capable of growing on GBT) has been shown to convert GBT and cobalamin to dimethylglycine (DMG) and methylcobalamin and was thus defined as a GBT methyltransferase (MtgB) (24). Similarly, an MtgB ortholog from *Methanolobus* sp. B1d was determined to transfer the GBT methyl group to coenzyme M, thereby playing a key role in GBT-dependent methylotrophic methanogenesis (25).

Most methanogens are incapable of efficiently converting GBT and choline to methane but first require the conversion of GBT or choline to TMA by bacteria. Coastal marine organisms typically accumulate GBT, with intracellular GBT being observed at concentrations of up to 1 M in some microorganisms living in hypersaline environments (26). Microcosm studies using coastal marine sediments have estimated that up to 90% of methane emitted from marine ecosystems can be produced from GBT and other structurally related quaternary amine compounds (17, 27). In addition, TMA *N*-oxide (TMAO) is an abundant compound in marine eukaryotic cells (28, 29) that is another potential precursor of TMA. Together, TMAO, GBT, and choline all can be converted to TMA by TMAO and dimethyl sulfoxide (DMSO) reductase (TorA and DMSOR), GBT reductase (GrdHI), or choline-TMA lyase (CutCD), respectively.

Molecular studies, including 16S rRNA gene diversity surveys and carbon-isotope coupled metagenome-assembled genome analysis, have shown that *Pelobacter* and *Methanococcoides* can syntrophically convert choline to methane in coastal saltmarsh sediments (30), while members of the novel family *Candidatus* “Betainaceae” fam. nov. that are affiliated with *Clostridiales* can convert GBT to TMA (26). In addition, the sulfur-reducing bacterium Desulfuromonas acetoxidans can degrade GBT to produce TMA and acetate via GBT reductase; it also converts GBT to methane in synergy with a methylotrophic methanogen (17). Nevertheless, the methanogenic archaea that produce methane and the bacterial taxa that convert GBT/choline to the methanogenic precursor TMA in cold seeps remain largely unknown.

In this study, GBT was used as the sole carbon source to obtain the Formosa cold seep microcosms, which efficiently converted GBT to methane. Species diversity and metagenomic analyses revealed the predominant methanogenic archaea and GBT-degrading bacterial species and the genes encoding GBT reductase (GrdH) and methyltransferase (MtgB) that are involved in the two primary anaerobic GBT degradation pathways, GBT reduction and demethylation, respectively. The isolated bacterial strain (ZWT) and methanogenic strain (LLY) both represent new species, respectively, and converted GBT to TMA and methane, and a reconstructed coculture of the two strains was able to convert GBT to methane at lower temperatures corresponding to *in situ* conditions. Remarkably, dimethylglycine (DMG), a GBT demethylation product, promoted growth of the two strains at lower temperatures, either in mono- or cocultures. Overall, this work determined that the widely used osmolyte GBT is the precursor for methane production in the Formosa cold seep and also of cryoprotectant for cold seep microorganisms.

## RESULTS

### Glycine betaine supports biogenic methane production in the Formosa cold seep.

**FIG 1:**
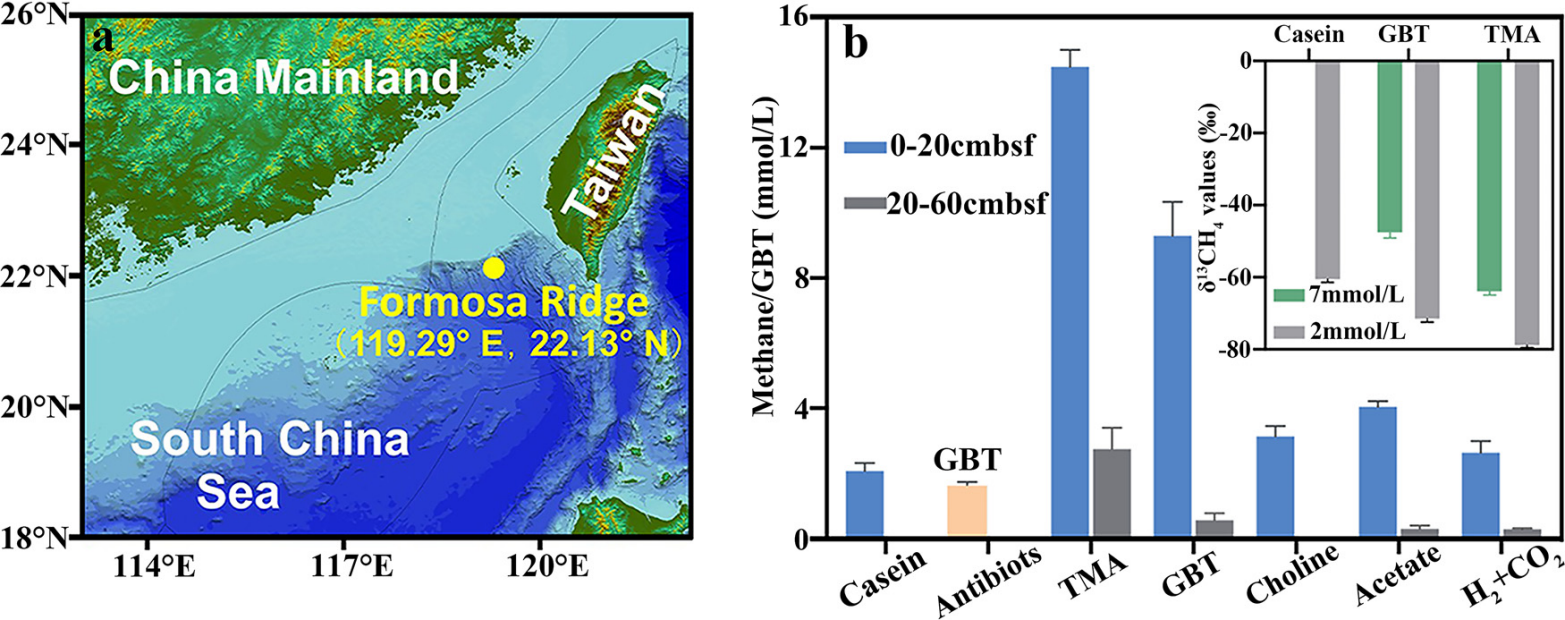
Biogenic methane precursor assays in the Formosa cold seep sediments. (a) Geographic location of the Formosa Ridge cold seep in the South China Sea where sediment samples were taken. The map was created using Global Mapper. (b) Each 0.5 g of sediment was sampled from either 0 to 20 cm below the sea floor (cmbsf) or from 20 to 60 cmbsf and dissolved in 5 ml of artificial seawater, followed by amendment with 0.25% casein and 1 mg/ml of ampicillin and kanamycin in addition to 20 mM trimethyl amine (TMA), glycine betaine (GBT), choline, acetate, or 0.1 MPa H_2_/CO_2_ (80:20). The sediment microcosms were incubated at 18°C, followed by measurement of methane and GBT accumulation (orange bar) after 9 weeks. The inset shows carbon isotope signatures of methane at two concentrations in microcosms amended only with casein, GBT, or TMA and were measured using isotope ratio MS. Triplicate microcosms of each tested substrate were evaluated, and the averages and standard deviations are shown.

To explore the microorganisms and metabolic pathways responsible for the biogenic methanogenesis in cold seeps, the reductive cold seep sediments at 1.165 m depth of the active site of Formosa Ridge (Fig. 1a; 22°06′89.05″N and 119°17′16.384″E) were sampled using a gravity core sampler during the Kexue 2019 expedition.

Cold seep sediment samples were used to establish methane production microcosms via inoculation into artificial seawater containing trace casein and amended either with methanogenic substrates or without substrates. After incubation at 18°C under N_2_ and CO_2_ headspaces (unless indicated otherwise), methane was produced from all of the sediment microcosms after 9 weeks of incubation. Six- and 4-fold higher methane contents were produced from the microcosms containing TMA and GBT than those from artificial seawater, but similar lower levels of methane were observed in those amended with choline, acetate, and H_2_ plus CO_2_ (Fig. 1b). This suggests that GBT and its reductive product, TMA, could be the major precursors for biogenic methanogenesis in the cold seep. In addition, more methane was produced from the upper sediment layer (0 to 20 cm below seafloor [cmbsf]) than the deeper layer (20 to 60 cmbsf), indicating that biogenic methane is mainly produced in the upper seafloor layer. To discern whether GBT-supported methanogenesis was mediated by methanogenic archaea alone or via a syntrophy of methanogenic archaea and bacteria in cold seep sediments, ampicillin and kanamycin were used to inhibit bacterial activities. Antibiotic amendment almost completely inhibited methane emissions, wherein only 0.05 mmol CH_4_/liter and 1.65 mmol/liter GBT were observed in the microcosm without addition of methanogenic precursors. Therefore, 97% of the methane production from GBT was estimated based on the 2.1 mmol CH_4_/liter emitted from the noninhibited microcosms. Antibiotic inhibition of methane production indicates that bacteria that presumably convert GBT to TMA synergistically with methanogenic archaea contribute to biogenic methanogenesis in cold seeps.

In addition, carbon isotope fractionation assays indicated that the sediment-released CH_4_ possessed a similar but slightly heavier carbon isotope signature (δ^13^C, −41.82 to −61.49%, depending on CH_4_ concentrations) than that from the GBT-amended microcosms (−47% to −71.56%) (Fig. 1b, inset). These values were much lighter than the δ^13^C of acetate-derived CH_4_ (−9% to −35%) and between CO_2_ reduction-produced methane (−28% to −79%). The carbon isotope fractionation values of the cold seep methane further suggested that biogenic methane in the system is most likely derived from GBT or its reductive product, TMA.

### Uncultured *Clostridiales* spp., *Methanococcoides* spp., and the genes involving in GBT metabolism are enriched in GBT-amended cold seep microcosms.

**FIG 2:**
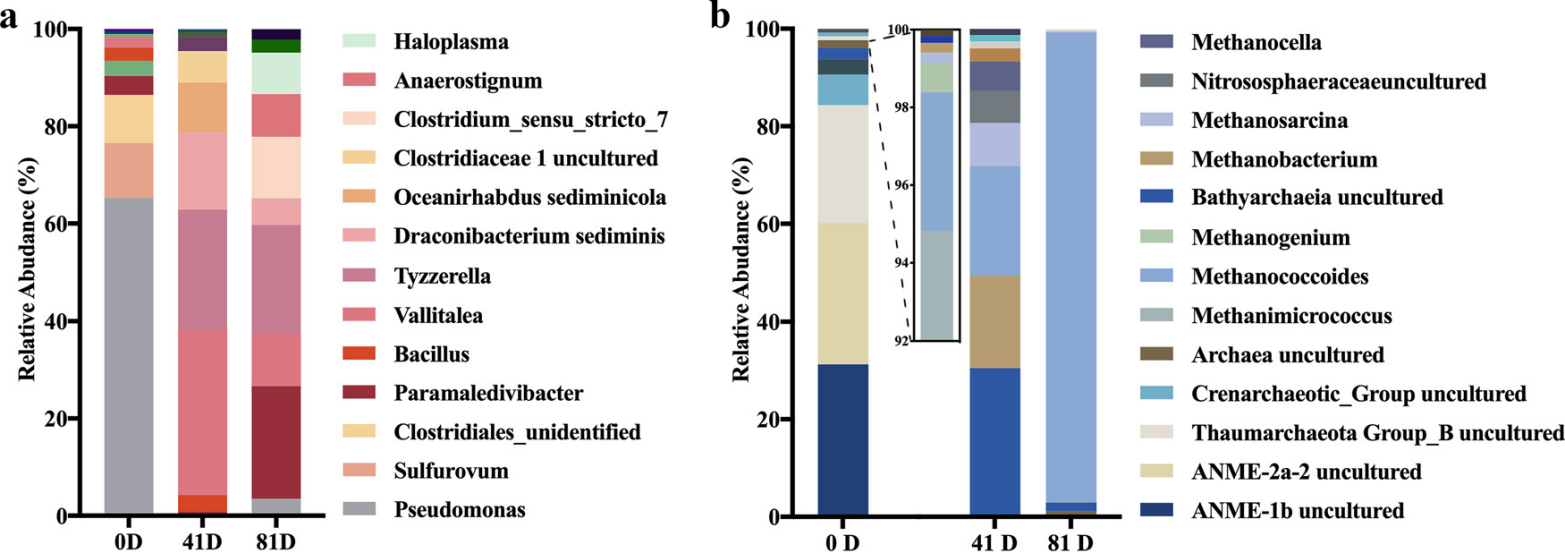
Dominant bacterial and archaeal genera assayed in GBT-enriched cold seep microcosms. Sediment (5 g) was inoculated into 50 ml of artificial seawater amended with 20 mM GBT, followed by incubation at 18°C. Total DNA was extracted at days 0, 41, and 81 of incubation and used to sequence the 16S rRNA V3-V4 hypervariable regions of both bacterial and archaeal genes. Bar diagrams indicate the relative abundances of the 10 most abundant bacterial (a) and archaeal (b) genera in the microcosms at three sampling times. The relative abundances of methanogenic archaeal genera at day 0 are shown in the zoomed-in diagram.

GBT was used as the sole carbon source to enrich the cold seep microorganisms that convert GBT to CH_4_. The upper layers from the seep sediments were incubated in 20 mM GBT for 81 days at 18°C, followed by measurement of CH_4_ production to identify GBT-dependent methanogenic microcosms. 16S rRNA gene diversity analyses revealed that the dominant bacterial genera in the GBT microcosms included *Vallitalea*, a Gram-negative halophile, *Tyzzerella* and *Oceanirhabdus* within the order *Clostridiales* (family *Clostridiaceae*), and the facultatively anaerobic *Draconibacterium.* In contrast, Pseudomonas, *Sulfurovum*, and unidentified *Clostridiales* were abundant in *in situ* sediments (Fig. 2a). Archaeal communities also markedly shifted after GBT enrichment, with dominant organisms becoming methanogens like *Methanococcoides* (<6% in sediment to 22.50% in microcosms) and *Methanobacterium* (<0.1% in sediment to 18.83% in microcosms) in addition to uncultured *Bathyarcheaia* (<0.1% in sediment to 30.49% in microcosms). In contrast, ANME-2a (28.91% in sediment) and ANME-1b (31.25% in sediment) groups and *Thaumarchaeota* group B (24.22% in sediments) were prevalent in *in situ* sediments but disappeared in the GBT enrichment after 7 weeks (Fig. 2b). After 81 days of incubation, the uncultured *Bathyarcheaia* also disappeared, and *Methanococcoides* increased to >90% relative abundance. The archaeal community shifts were consistent with methane production in the microcosms.

**FIG 3:**
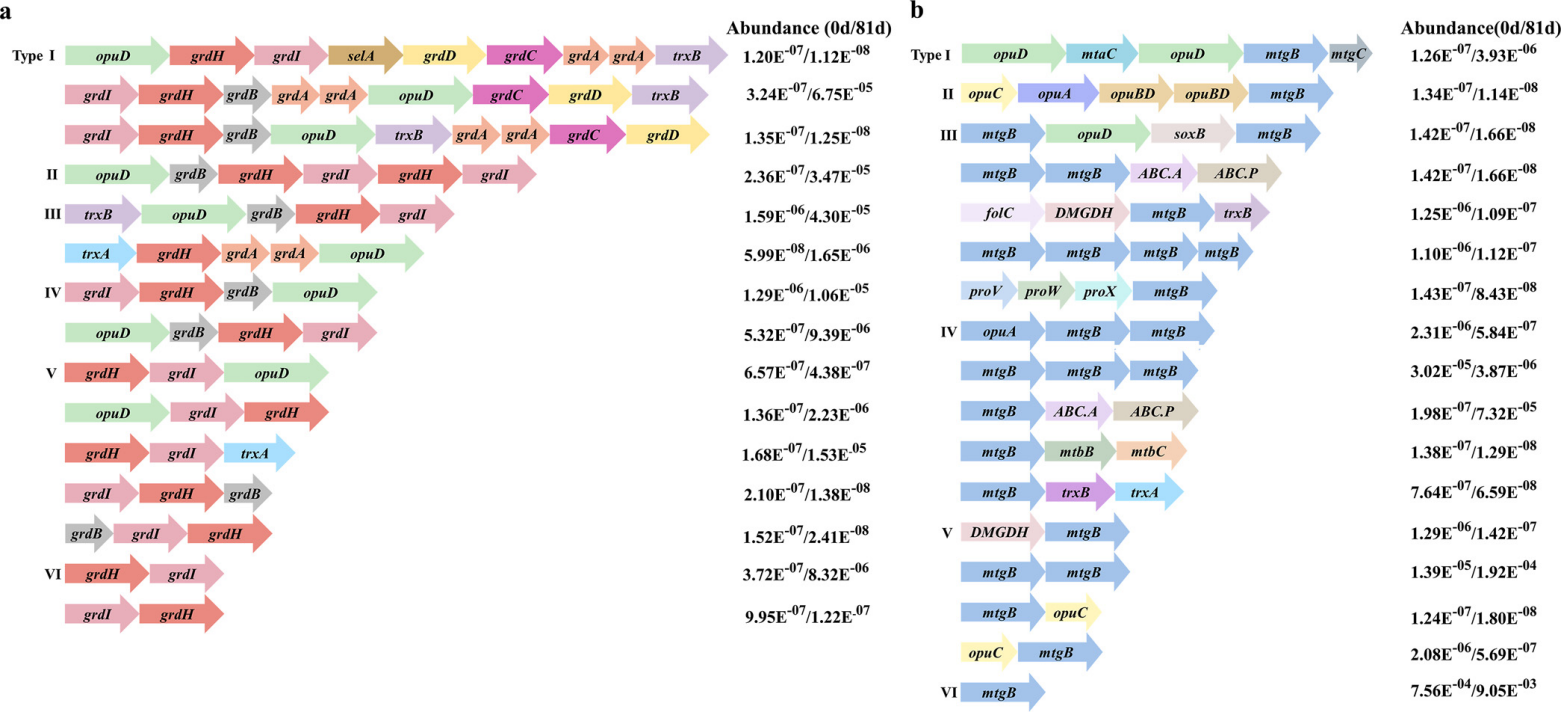
Genes involved in GBT reduction and demethylation in metagenomic contigs retrieved from *in situ* sediments and GBT-enriched cold seep microcosms. Total DNA extracted from day 0 and 81 incubations of the GBT microcosms were used for metagenomic sequencing and contig assembly. Functional annotations were predicted using KofamKOALA (50), and the hit scores above predefined thresholds of 565.60 for *grdH* (K21579), 108.87 for *mtgB* (K14083), and 663.63 for *opuD* (K05020) were defined, respectively. According to gene contents and arrangements, gene cluster types were defined. Relative abundances of genes involved in GBT reduction (a) and demethylation (b) among the total sequence reads that are defined as one are shown. *grdA*, glycine/sarcosine/GBT reductase complex component A; *grdB*, glycine reductase complex component B subunit gamma; *grdC*, glycine/sarcosine/GBT reductase complex component C subunit beta; *grdD*, glycine/sarcosine/betaine reductase complex component C subunit alpha; *grdE*, glycine reductase complex component B subunits alpha and beta; *grdH*, GBT reductase complex component B subunit beta; *grdI*, GBT reductase complex component B subunit alpha; *opuD*, GBT transporter; *trxB*, thioredoxin reductase; *trxA*, thioredoxin; *selA*, l-seryl-tRNA(Ser) selenium transferase; *mtaC*, methanol corrinoid protein; *mtgB*, GBT corrinoid protein, CoM methyltransferase; *mtgC*, GBT corrinoid protein; *proV*, GBT/proline transport system ATP-binding protein; *opuC*, osmoprotectant transport system substrate-binding protein; *opuBD*, osmoprotectant transport system permease protein; *opuA*, osmoprotectant transport system ATP-binding protein; soxB, sarcosine oxidase subunit beta; *proW*, GBT/proline transport system permease protein; *proX*, GBT/proline transport system substrate-binding protein; *ABC.A*, putative ABC transport system ATP-binding protein; *ABC.P*, putative ABC transport system permease; *mtbB*, dimethylamine corrinoid protein CoM-methyltransferase; *mtbC*, dimethylamine corrinoid protein; *DMGDH*, dimethylglycine dehydrogenase; *folC*, dihydrofolate synthase/folylpolyglutamate synthase.

Metagenomic analyses were then conducted on cold seep sediments and GBT microcosms using Illumina HiSeq-based shotgun metagenomic sequencing. The assembled contigs were used for functional annotations, and proteins pertinent with GBT reduction (GrdH and GrdB) and its transporter (OpuD) were further analyzed for phylogenetic clustering with the respective functionally defined proteins (Fig. S1 in the supplemental material). In both the *in situ* sediments and GBT microcosms, genes or gene clusters involved in the two major GBT anaerobic degradation pathways, GBT reduction (Fig. 3a) and GBT demethylation (Fig. 3b), were enriched. Genes involved in the GBT reduction pathway included *grdHI*, which encodes GBT reductase complex B that specifically reduces GBT to TMA, and acetyl phosphate, *opuD*, which encodes a transporter for GBT, and the structurally analogous *grdABCD* encoding a reductase complex that reduces glycine, sarcosine, or GBT. In addition, genes encoding thioredoxin and its reductase that could provide reducing equivalents to GBT reduction were also detected. Overall, 26 contigs possessing *grdHI* were obtained, and these genes were organized into 6 cluster types (Fig. 3a). Of these contigs, some (like those in types I, III, and V) were enriched by about 100-fold in GBT microcosms. In addition, 503 contigs carrying the *mtgB* gene cluster, which is likely involved in GBT demethylation, were identified. It is worth noting that *mtgB* (encoding the GBT corrinoid protein-CoM methyltransferase) and *mtgC* (encoding the GBT corrinoid protein) were clustered with more versatile GBT transporters than those clustered with *grdHI* genes. In addition to *opuD* clustering with *grdHI*, *opuABC* (encoding an osmoprotectant transport system), and another two potential GBT transporters (*proWX* encoding GBT/proline transporters and ABC.AP encoding ABC transporters) were also identified. The identified *mtgB* genes were also organized as six cluster types, with types I, IV, V, and VI being enriched by 10- to nearly 300-fold (Fig. 3b). This suggests that two GBT metabolic processes, GBT reduction to TMA and demethylation to dimethylglycine, could co-occur in the cold seep environments. In contrast, genes encoding TMAO reductase (TorA) or choline-TMA lyase (CutCD) were not found in either the *in situ* sediments or GBT microcosms, thereby rendering TMA production from either TMAO or choline unlikely.

10.1128/mSystems.00703-21.1 FIG S1 Phylogenetic clustering analysis of the glycine betaine proteins retrieved from Formosa cold seep and Gulf of Mexico methane seep. Functional annotations of the ORFs from Formosa cold seep were predicted using KofamKOALA (50), and hit scores above predefined thresholds were selected as follows: 565.60 for GBT reductase subunit gene *grdH* (K21579), 94.57 for glycine reductase subunit gene *grdB* (K10672), and 663.63 for GBT transporter gene *opuD* (K05020). The GrdH sequences from the Gulf of Mexico were downloaded from the IMG/M database under accession numbers of 3300008340 and 3300009874 (cold seep region) and 3300008416 and 3300008417 (noncold seep), respectively. Obtained protein sequences were aligned with the corresponding reference sequences using MAFFT v7.407 (parameter, --auto). Alignments were trimmed using trimAl 1.2rev59 (parameter, -noallgaps). The maximum-likelihood trees were constructed with IQ-TREE v2.0.3 (parameters, -bb 1000 -m TEST -nt AUTO), and the statistical supports of trees were obtained by bootstrapping 1,000 iterations. The resulting trees were visualized and edited using iTOL v5. (a) Phylogenetic clustering of GrdH and GrdB. In total, 39 GrdH and 222 GrdB sequences from Formosa cold seep (prefixed with F) and 75 GrdH sequences from the Gulf of Mexico methane seep (prefixed with M) and 7 reference sequences were aligned. (b) Phylogenetic clustering of OpuD. Fifty-six OpuD sequences retrieved from Formosa cold seep were aligned with 5 reference sequences downloaded from the NCBI database. Download FIG S1, TIF file, 1.8 MB. Copyright © 2021 Li et al. 2021 Li et al. https://creativecommons.org/licenses/by/4.0/ This content is distributed under the terms of the Creative Commons Attribution 4.0 International license.

Sequence homology analysis indicated that the 13 types of identified *grdH* were associated with numerous organisms, with 38 *grdH* orthologs detected in the cold seep primarily associated with unclassified *Firmicutes*, *Proteobacteria*, *Spirochaetia*, and *Fusobacteriia* (Fig. S2a). The 503 enriched orthologs were mainly associated with *Firmicutes*, *Chloroflexi*, *Proteobacteria*, and *Planctomycetes* (Fig. S2b), while those from *Euryarchaeota* were underrepresented. It is worth noting that the MtgB phylogeny was not topologically congruent with that for the species carrying the corresponding MtgB orthologs, suggesting the presence of frequent horizontal gene transfer in cold seep sediment systems. Given that only a small portion of the cold retrieved *mtgB* from methanogenic archaea, we predicted that methanogen-mediated GBT-derived methanogenesis could have little contribution to the cold seep biogenic methane.

10.1128/mSystems.00703-21.2 FIG S2 Analysis of the phylogenetic affiliations of the *grdH* (a) and *mtgB* (b) genes enriched from Formosa cold seep that involve in GBT reduction and demethylation pathways, respectively. Functional annotations for ORFs were predicted using KofamKOALA (50), and hit scores above predefined thresholds of *grdH* (K21579) of 565.60 and mtgB (K14083) of 108.87, respectively, of each KO were selected. GrdH and MtgB amino acid sequences from the known species that are deposited in the NCBI database (black lines) and those retrieved from Formosa cold seep metagenome contigs (blue lines) were aligned with the MUSCLE algorithm. The neighbor-joining trees of GrdH and MtgB proteins were constructed in MegaX, and the statistical supports of trees were obtained by bootstrapping 1,000 iterations. The resulting trees were edited and visualized with iTOL. Download FIG S2, TIF file, 0.9 MB. Copyright © 2021 Li et al. 2021 Li et al. https://creativecommons.org/licenses/by/4.0/ This content is distributed under the terms of the Creative Commons Attribution 4.0 International license.

### Oceanirhabdus seepicola sp. nov., a novel bacterium isolated from cold seep microcosms, reduces GBT to TMA at 18°C.

**FIG 4:**
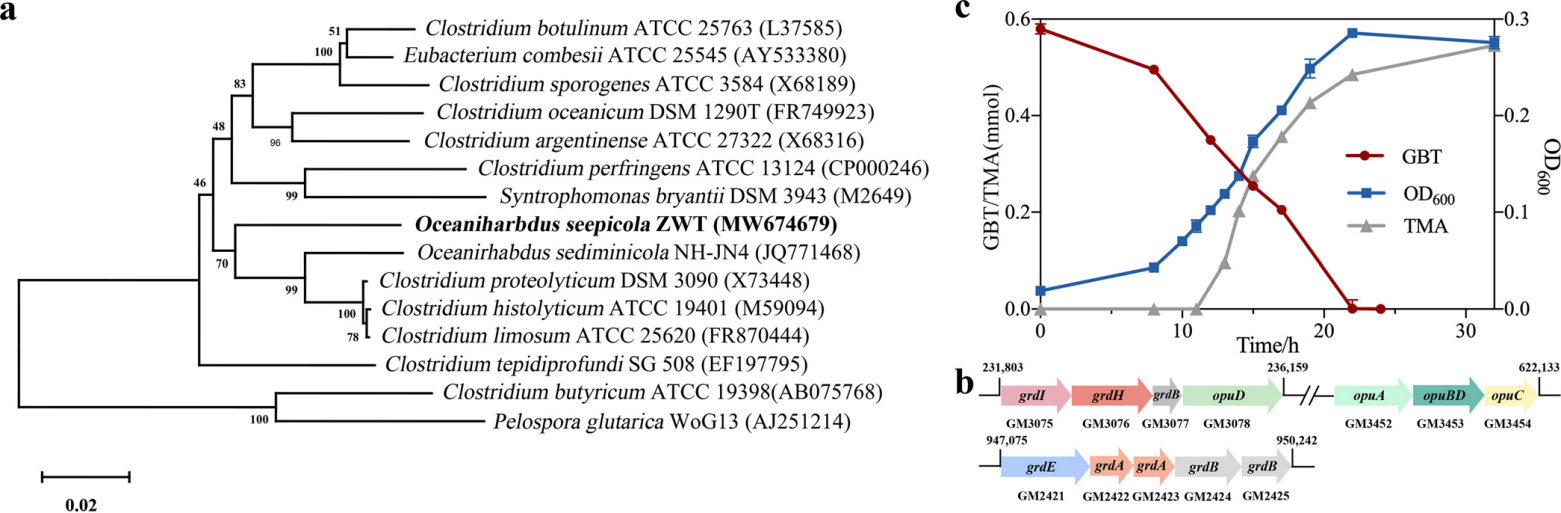
Oceanirhabdus seepicola sp. nov. isolated from the GBT microcosms of cold seep encodes a GBT reductase gene cluster and efficiently reduces GBT to TMA at 18°C. (a) Neighbor-joining phylogenetic tree based on 16S rRNA genes of strain ZWT and its closest relatives. GenBank accession numbers are given in parentheses. Percentages of bootstrap support are shown at branch nodes. Scale bar indicates 2% sequence divergence. (b) Gene clusters involved in GBT reduction identified in the strain ZWT genome. Gene annotations are the same as in Fig. 3a, and gene numbers are shown underneath individual genes. The genome locations of the genes involved in GBT reduction are also shown. (c) Strain ZWT was grown in 20 mM GBT at 18°C, and growth was monitored by measuring OD_600_ values over 1-h intervals. In addition, GBT and TMA contents in cultures were measured using high-performance liquid chromatography (HPLC). Three culture replicates were assayed for each measurement and the averages and standard deviations are shown.

The bacterial strain ZWT was isolated from GBT-enriched cold seep microcosm at day 41 using GBT as the sole carbon source. Strain ZWT converted 20 mM GBT to TMA stoichiometrically, in addition to using glycine, glucose, and maltose as sole carbon sources (Table S1). Strain ZWT exhibited the highest 16S rRNA sequence identity (94%) with Oceanirhabdus sediminicola NH-JN4 (Fig. 4a) within the *Clostridiaceae* family. Thus, strain ZWT was identified as a novel species of the genus *Oceanirhabdus* as Oceanirhabdus seepicola sp. nov. for noting a seep inhabitant and was deposited in the China General Microbiological Culture Collection Center under accession no. CGMCC 1.17897 and the Japan Collection of Microorganisms under accession no. JCM 34232.

10.1128/mSystems.00703-21.4 TABLE S1 Differential phenotypic and physiological characteristics of strain ZWT and its closest phylogenetic relative. Download Table S1, DOCX file, 0.01 MB. Copyright © 2021 Li et al. 2021 Li et al. https://creativecommons.org/licenses/by/4.0/ This content is distributed under the terms of the Creative Commons Attribution 4.0 International license.

The genome of Oceanirhabdus seepicola sp. nov. (JAGSOJ000000000; Table S2) possesses two gene clusters encoding GBT reductases (Fig. 4b). One cluster encodes the GBT-specific reductase complex B, including *grdI*, *grdH*, and *grdB* that encode the alpha, beta, and gamma subunits and *opuABDC* that encodes the GBT transporter. The other cluster comprises *grdE* and *grdAB* that encode glycine/sarcosine/GBT reductase complex components A and B. In addition, genes encode trimethylamine permease (GM000616, GM002195, and GM002197) were found in the genome. Although the genome contains a gene encoding the choline-TMA lyase-activating enzyme, the gene encoding choline-TMA lyase (CutC) was not identified. Therefore, strain ZWT carries the GBT reductase genes that were detected in the cold seep metagenomes.

10.1128/mSystems.00703-21.5 TABLE S2 Genome statistics. Download Table S2, DOCX file, 0.01 MB. Copyright © 2021 Li et al. 2021 Li et al. https://creativecommons.org/licenses/by/4.0/ This content is distributed under the terms of the Creative Commons Attribution 4.0 International license.

Consistent with the genomic analyses, O. seepicola ZWT can use GBT as the sole carbon source, which supported a growth rate of 0.189 per hour, but it also converts GBT to equimolecular concentrations of TMA at a degradation rate of 0.01 mmol·h^−1^ at 18°C (Fig. 4c). In addition, O. seepicola ZWT can grow with casein as the sole carbon and nitrogen sources, but not choline. Compared to Oceanirhabdus sediminicola NH-JN4, O. seepicola ZWT grew at a lower temperature range (4 to 30°C), and optimally between 18 to 22°C, and was able to use GBT and glucose (Table S1). Therefore, the distinct phenotypic characteristics of strain ZWT also supported its independent species status along with lower 16S rRNA sequence homology.

### Methanococcoides seepicolus sp. nov. isolated from the GBT microcosm produces methane from TMA and through GBT demethylation.

**FIG 5:**
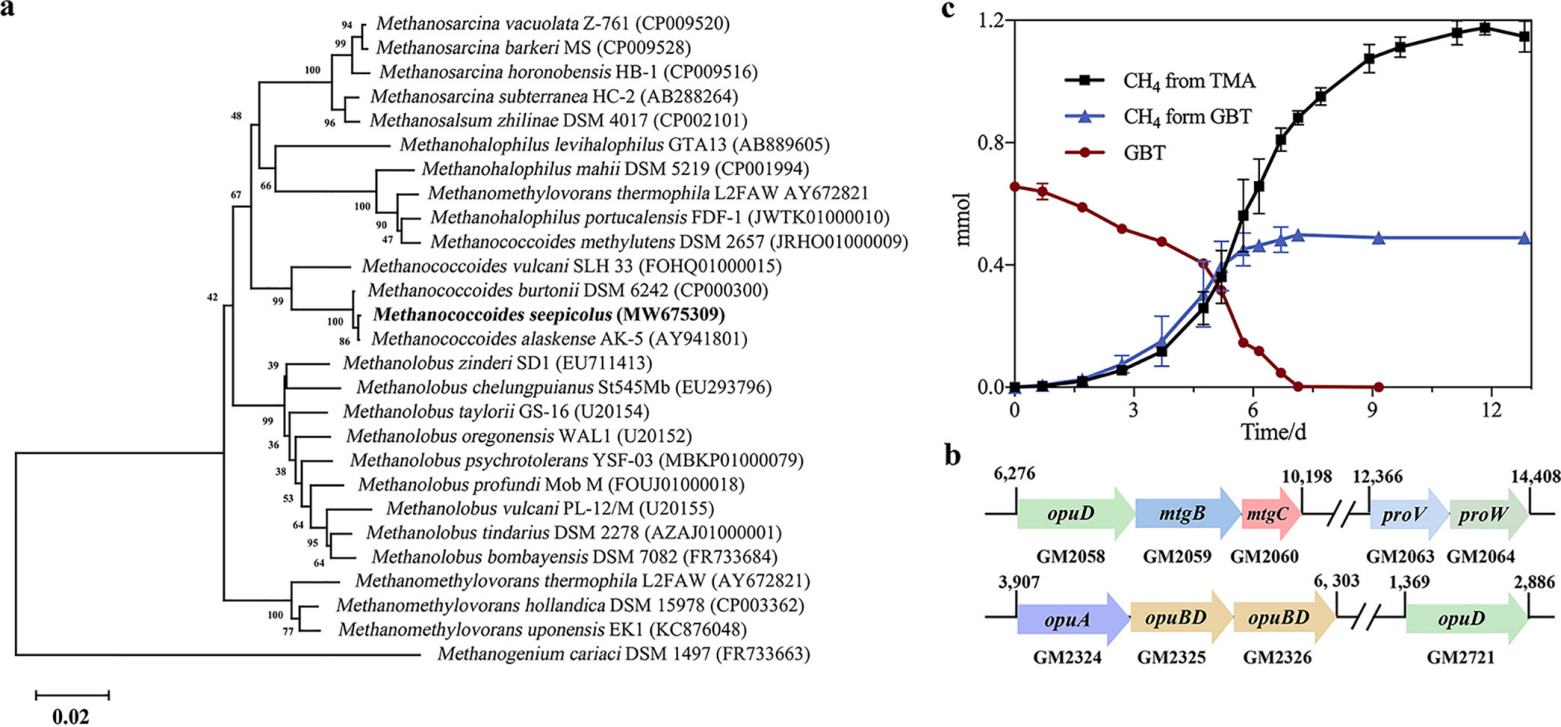
Methanococcoides seepicolus sp. nov., isolated from the GBT microcosms, encodes genes involved in GBT demethylation and converts GBT to methane at 18°C. (a) Neighbor-joining phylogenetic tree based on the 16S rRNA gene sequence of strain LLY and those from its closest relatives. GenBank accession numbers are given in parentheses. Percentages of bootstrap support are shown at branch nodes. Scale bar indicates 2% sequence divergence. (b) Gene clusters involved in GBT demethylation identified in the strain LLY genome. Gene annotations are the same as in Fig. 3b, and gene numbers are shown underneath gene designations. The genome locations of GBT demethylation genes are also shown. (c) M. seepicolus was grown in 20 mM GBT at 18°C, and growth was monitored by measuring methane production over 1-day intervals. In addition, GBT in cultures and CH_4_ in headspaces were measured using HPLC and GC, respectively. Three culture replicates were assayed for measurements, and the averages and standard deviations are shown.

A methanogenic archaeon was also isolated from the GBT microcosm developed from cold seep sediments at day 41. A methane-producing cocci-like strain LLY was obtained that used GBT as its sole carbon source. Strain LLY (CGMCC 1.17896) exhibited 99% 16S rRNA gene similarity to that of Methanococcoides alaskenses and Methanococcoides burtonii (Fig. 5a) and was thus identified as a member of the genus *Methanococcoides*. The complete genome of *Methanococcoides* LLY (JAGSOI000000000; Table S2) was then sequenced, which exhibited an average nucleotide identity (ANI) of 91% to the genome of M. burtonii, while a genome for Methanococcoides alaskenses is unavailable. Therefore, the novel species Methanococcoides seepicolus sp. nov. for noting a seep inhabitant was proposed for strain LLY.

The M. seepicolus genome contained the entire gene suite involved in methylotrophic methanogenesis, in addition to those pertinent to methanogenesis from GBT, which include *mtgB* and *mtgC* that encode GBT corrinoid protein-coenzyme M methyltransferase and GBT corrinoid protein, respectively, as well as the gene encoding the BCCT family of glycine/GBT ABC transporter permeases. The MtgBC protein complex catalyzes the transfer of the GBT methyl group to CoM, which is then reduced to methane by CoM reductase. In addition, GBT is demethylated to dimethylglycine (DMG). Genes encoding GBT or choline ABC transporters were also identified in the genome (Fig. 5b).

Unlike M. alaskenses and M. burtonii, M. seepicolus LLY can grow on and convert 1 mol of GBT to roughly 0.75 mol of methane (Fig. 5c), in contrast to 3 mols CH_4_ produced per mol of TMA. This suggests that strain LLY only uses one of the three methyl groups of GBT to produce CH_4_ via the GBT demethylation pathway, and it is consistent with the GBT methyltransferase MtgBC genes in the genome. In contrast, a lower methanogenesis rate was identified when using GBT compared to that from TMA (Fig. 5c), suggesting that TMA is the preferential substrate of M. seepicolus.

In addition to GBT and TMA, M. seepicolus also used methanol, but not choline, to produce methane. Cells grew over a temperature range of 0 to 25°C, and optimally at 18°C, which differed from growth characteristics of M. alaskenses and M. burtonii and confirmed the distinct species status (Table S3) along with the observed lower-genome ANI.

10.1128/mSystems.00703-21.6 TABLE S3 Differential phenotypic and physiological characteristics of strain LLY and its closest phylogenetic relatives. Download Table S3, DOCX file, 0.01 MB. Copyright © 2021 Li et al. 2021 Li et al. https://creativecommons.org/licenses/by/4.0/ This content is distributed under the terms of the Creative Commons Attribution 4.0 International license.

### Oceanirhabdus seepicola and Methanococcoides seepicolus syntrophically convert GBT to methane.

**FIG 6:**
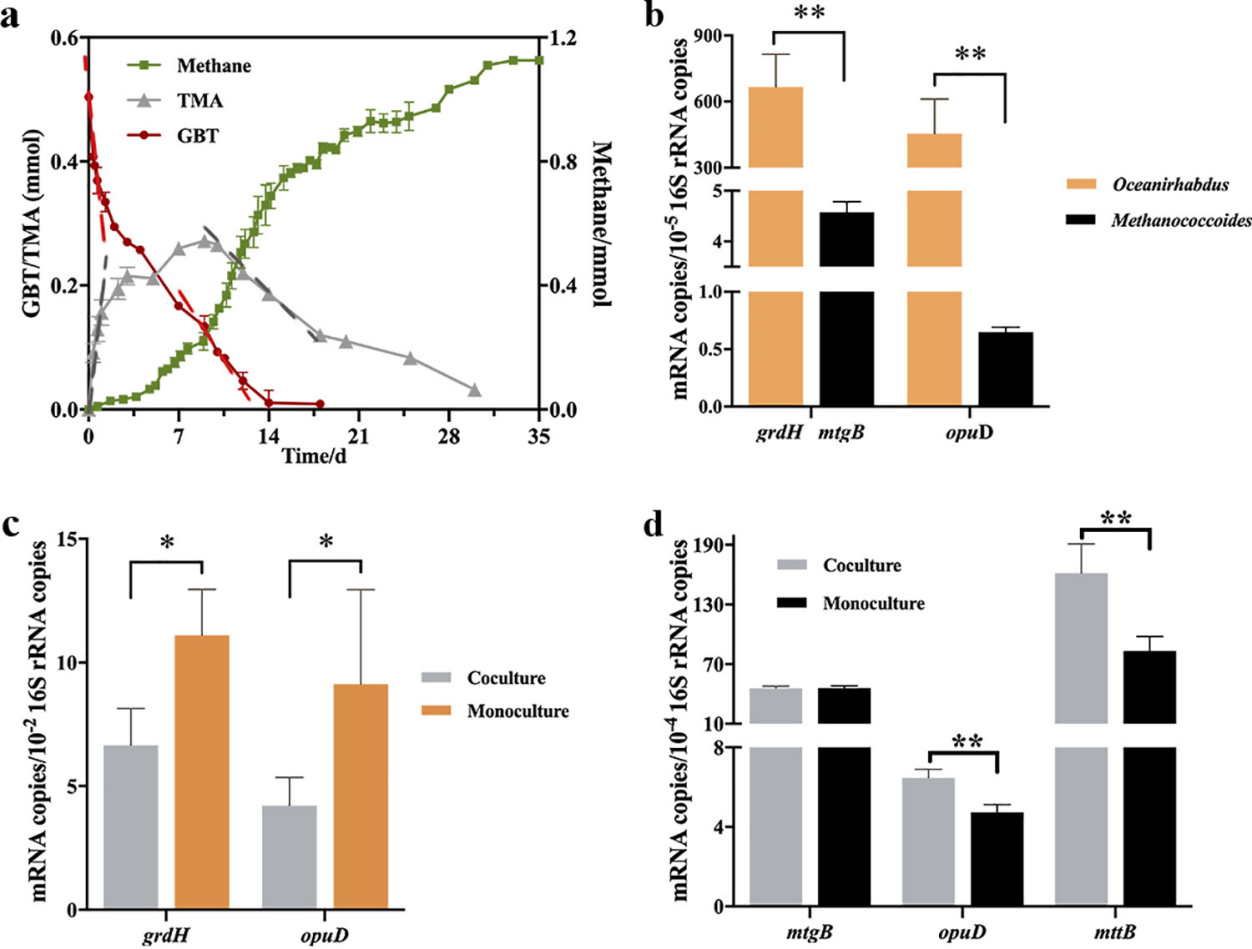
The coculture of Oceanirhabdus seepicola and Methanococcoides seepicolus converts GBT to CH_4_ at 18°C, and associated genes are expressed. (a) Mid-exponential cultures of O. seepicola and M. seepicolus were inoculated (each 10%) into a basal medium containing 20 mM GBT and incubated at 18°C. Methane production, GBT degradation, and TMA accumulation were monitored via GC and HPLC assays during cultivation, while production and degradation rates of the three substances were calculated based on linear regression plots (dotted lines). (b, c, and d) Quantification of mRNA abundances of the genes involved in GBT conversion to methane in coculture based on quantitative RT-PCR. 16S rRNA gene copy numbers of the two strains were measured for normalization to cell abundances. (b) Total RNA was extracted from cocultures during exponential growth phases of O. seepicola (day 3) and M. seepicolus (day 12), followed by measurement of transcript abundances of O. seepicola *grdH* and *oupD*, in addition to M. seepicolus *mtgB* and *oupD*. (c) Comparison of transcript abundances of O. seepicola *grdH* and *oupD* in coculture (day 3) and O. seepicola monococultures (15 h). (d) Comparison of M. seepicolus *mtgB*, *oup*D, and *mttB* expression levels in coculture (day 12) and in M. seepicolus monococulture (day 5). Three replicate cultures were used for measurements and triplicate mRNA quantifications for each measurement. The averages and standard deviations are shown.

To investigate how the two cold seep isolates synergistically convert GBT to methane, 10% of the mid-exponential cultures for each of O. seepicola and M. seepicolus were inoculated into basal medium containing 20 mM (0.45 mmol) GBT as the sole carbon source. After 15 days of incubation at 18°C, GBT was completely degraded at rates of 0.24 mmol · day^−1^ at day 1 and at 0.025 mmol · day^−1^ between days 2 and 15. TMA concentrations also peaked in the first day and then decreased to 0.05 mmol at a rate of 0.02 mmol · day^−1^. Methane production initiated at day 2 and continued until reaching 1.1 mmol at a rate of 0.097 mmol · day^−1^, and with a final CH_4_ yield of about 2.8 per 1 GBT degraded (Fig. 6a). This indicates that GBT is reduced by O. seepicola to TMA but not by *Methanococcoides* via GBT demethylation, while the TMA is then converted to CH_4_ by M. seepicolus. It is worth noting that cocultures exhibited lower rates of either GBT degradation or methane production than monococultures. Thus, inhibiting factors were likely present in the coculture.

We then compared expressions of the key genes involved in GBT metabolism in monococultures and in coculture. Total RNA was extracted from cocultures and GBT monococultures of O. seepicola and M. seepicolus at the respective mid-exponential phases, followed by estimation of transcript abundances for corresponding genes via quantitative reverse transcription-PCR (RT-PCR). It was found that the O. seepicola *grdH* exhibited about 60-fold higher expression levels than M. seepicolus *mtgB* in coculture (Fig. 6b). Similarly, O. seepicola *opuD* (encodes a GBT transporter) exhibited about 1,000-fold higher transcription than the archaeal ortholog. In contrast, the O. seepicola *grdH* and *opuD* all exhibited decreased transcriptional expression in coculture compared to in monococulture (Fig. 6c), suggesting that they were suppressed by unknown factors in the coculture.

M. seepicolus *mtgB* maintained similar expression levels in the mono- as in cocultures, while *mttB* (encoding trimethyltransferase) was increasingly expressed in the cocultures compared to the monococultures growing in GBT (Fig. 6d). Collectively, the gene expression profiles support that O. seepicola is the major player in GBT metabolism within the coculture by catabolizing GBT reduction to TMA, while M. seepicolus converts TMA to methane instead of demethylating GBT to dimethylglycine, most likely attributed to the lower competitiveness of the archaeon for attaining GBT when in coculture with the bacterial partner.

### The GBT demethylation product dimethyl glycine acts as a cold protectant for both the bacterium and archaeon.

**FIG 7:**
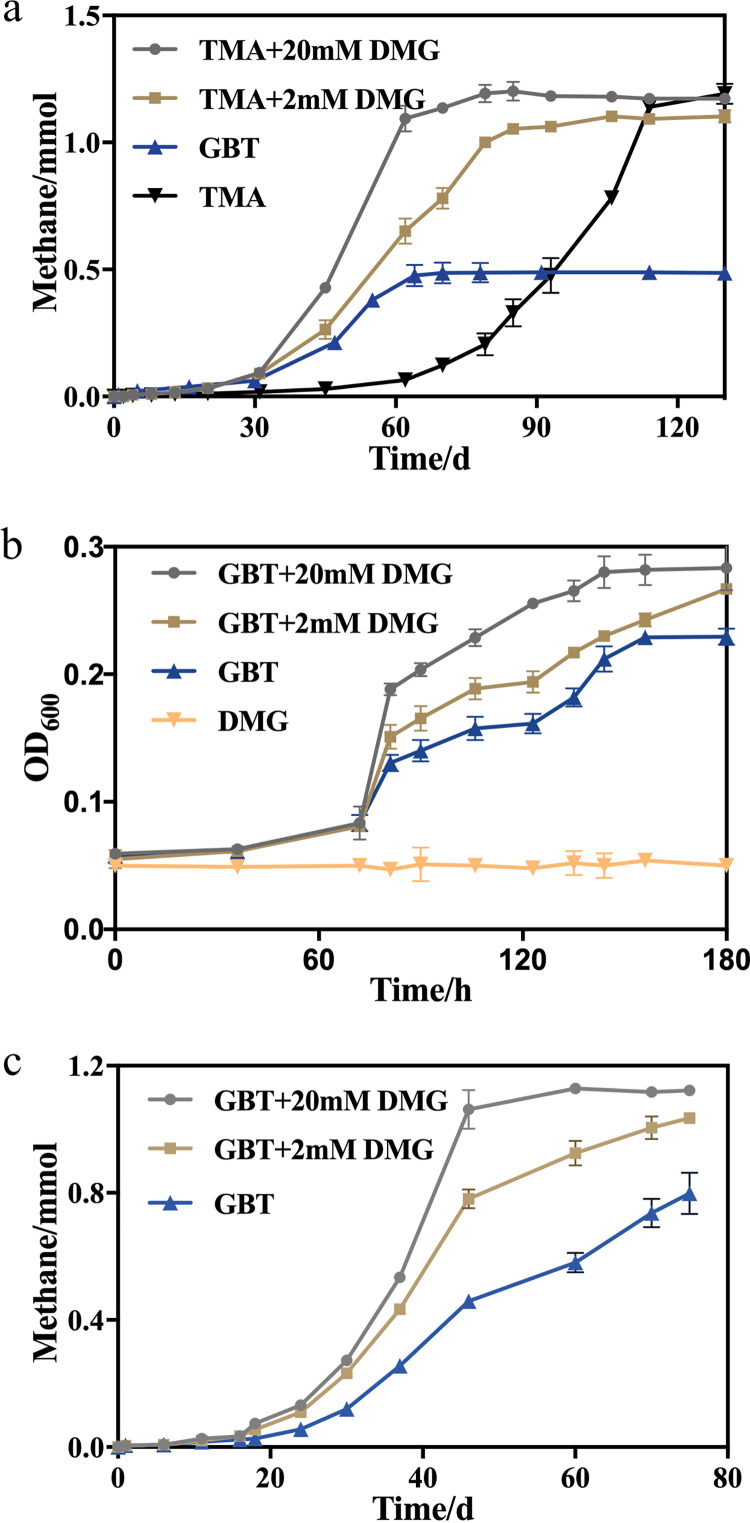
Dimethylglycine promotes the growth of both Methanococcoides seepicolus and Oceanirhabdus seepicola in mono- and cocultures at 4°C. Mid-exponential cultures of M. seepicolus and O. seepicola were inoculated at 10% into YTG basal medium containing 20 mM GBT or TMA (a) and 20 mM GBT or DMG (b), and 10% of each into GBT medium to develop a coculture (c), respectively. Dimethylglycine (DMG) at final concentrations of 2 mM and 20 mM were, respectively, dosed to the three cultures. Mono- and cocultures were grown at 4°C and 18°C (Fig. S2), respectively. Methane production (a and c) and OD_600_ (b) were measured during incubation, respectively. Rates of methane production and growth were calculated from the linear portions of the curves. Triplicate cultures were assayed, and the averages and standard deviations are shown.

To mimic *in situ* temperatures, M. seepicolus was grown at 4°C with GBT and TMA individually. Unexpectedly, though higher methane production rates were observed from TMA (0.03 mmol · day^−1^) than from GBT (0.01 mmol · day^−1^), a much longer lag time (25 days) was observed for the TMA culture than the GBT culture (5 days) (Fig. 7a), while the lag time difference when grown on the two substrates was not observed for 18°C cultures (Fig. 5c). Given that GBT demethylation will produce dimethylglycine (DMG), which is a cold protectant for *Bacillus* (31, 32), DMG was amended into the TMA culture. Dosing at 2 mM or 20 mM DMG greatly shortened the 4°C TMA culture lag phase of M. seepicolus but did not elevate the methane production rate (Fig. 7a), while DMG dosing did not affect the 18°C culture (Fig. S3a). Notably, DMG dosing did not increase the final methane yield of the TMA culture, indicating that DMG acts only as a cold protectant but not a methanogenic substrate and can be accumulated during GBT demethylation by *Methanococcoides*.

10.1128/mSystems.00703-21.3 FIG S3 Assay of dosing dimethylglycine in promoting 18°C growths of Methanococcoides seepicolus and Oceanirhabdus seepicola in either mono- or cocultures. The mid-exponential cultures of M. seepicolus and O. seepicola were 10% inoculated into the YTG basal medium containing 20 mM of GBT or TMA (a), and 20 mM of GBT or dimethylglycine (DMG) (b), and each 10% was added into GBT culture to setup a coculture (c), respectively. DMG at final concentrations of 2 mM and 20 mM were dosed to the three cultures. The mono- and cocultures were grown at 18°C. Methane productions (a and c) and OD_600_ (b) were followed during incubation, respectively. Triplicate cultures were assayed, and the averages and standard deviations are shown. Download FIG S3, TIF file, 0.4 MB. Copyright © 2021 Li et al. 2021 Li et al. https://creativecommons.org/licenses/by/4.0/ This content is distributed under the terms of the Creative Commons Attribution 4.0 International license.

DMG not only promoted the growth rate of the O. seepicola in GBT at 4°C but also the final cell yields (Fig. 7b), likely by elevating the GBT utilization, as DMG was not used as a carbon source. Remarkably, dosing 2 mmol and 20 mmol of DMG to 4°C cocultures led to the GBT-derived methane production rate being elevated from 0.02 mmol · day^−1^ to 0.035 mmol · day^−1^ and 0.05 mmol · day^−1^, respectively (Fig. 7c). In contrast, DMG dosing did not promote the GBT culture of O. seepicola (Fig. S3b) nor the coculture in methane production rates at 18°C (Fig. S3c). Consequently, these experimental results demonstrate that DMG acts as a cold protectant for both the cold seep bacteria and archaea. Therefore, the widely distributed osmoprotectant GBT could play dual roles by acting as the major precursor for methane production in addition to the cold protectant DMG in cold seep communities.

## DISCUSSION

**FIG 8:**
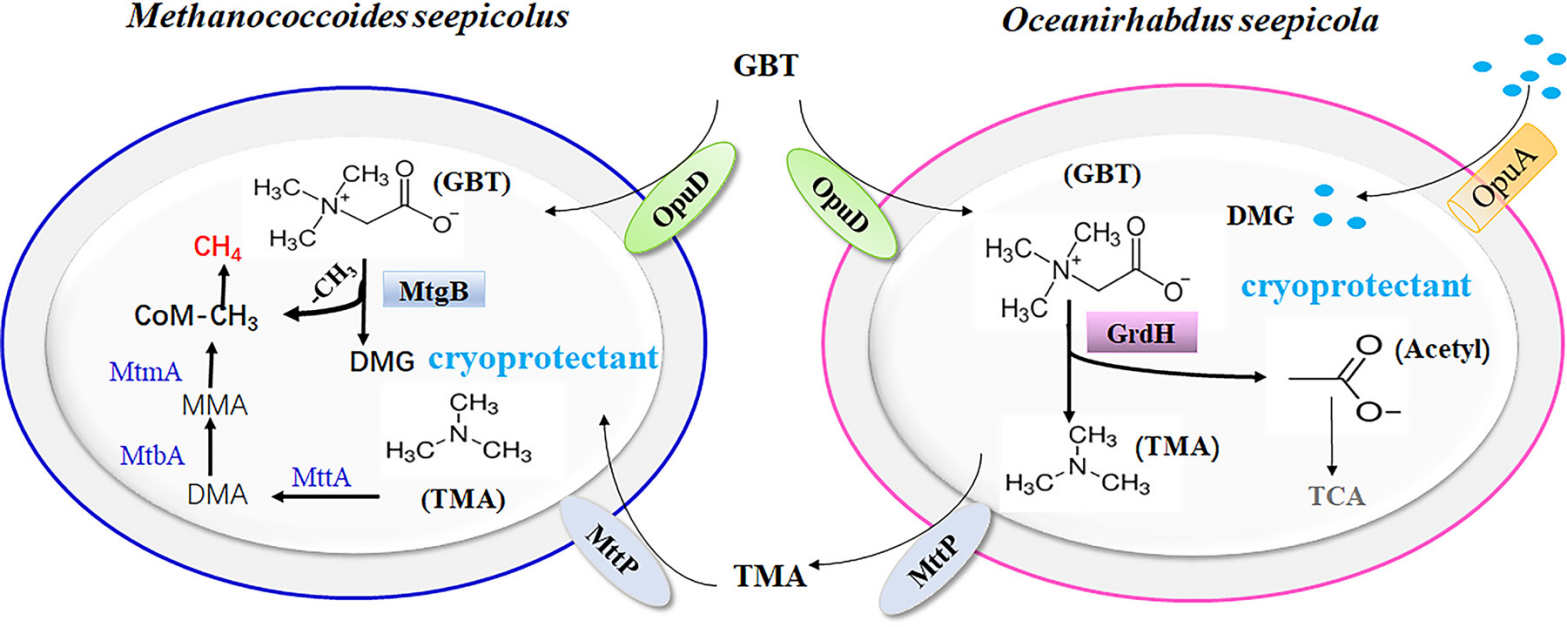
Schematic depicting the syntrophy between bacteria and methanogenic archaea in the conversion of GBT to methane in cold seep sediments. GBT is a widely used osmoprotectant and is reduced to trimethylamine (TMA) and acetyl-CoA via the bacterial GBT reductase complex (represented by GrdHI). TMA is then released from bacteria (pink cell) and imported by methanogens (blue cell) via the TMA transporter (MttP), followed by conversion to CH_4_ through the methylotrophic methanogenic pathway. GBT is also demethylated to dimethylglycine (DMG) and a methyl group (−CH_3_). The latter is converted to CH_4_, while DMG is used as a cryoprotectant (light blue dots) within cold seep bacterial and methanogenic archaeal cells.

Methane seeps are globally widespread seafloor ecosystems wherein methane gas is abundant. Chemical studies have recently indicated that biogenic methane comprises a substantial portion of cold seep methane, like in the Sonora Margin and SCS cold seeps (11, 19). In this study, detection of methane emissions from cold seep microcosms amended with various substrates indicated that GBT was the primary methanogenic precursor. Investigation and identification of the predominant species and metabolic genes within the SCS Formosa cold seep communities and GBT-amended microcosms also indicated that GBT reduction and demethylation were predicted as the primary pathways underlying GBT-derived methane in the cold seep. The bacterium Oceanirhabdus seepicola and the methanogenic archaeon Methanococcoides seepicolus were isolated from cold seep GBT microcosms, and both represent novel species of psychrophiles that grew optimally at 18°C. The former efficiently reduced GBT to TMA via the GBT reduction pathway, while the latter converted both TMA and GBT to CH_4_ via the GBT demethylation pathway. The two species were cocultured and stoichiometrically converted GBT to methane. Remarkably, the demethylated GBT product, DMG, functioned as a cryoprotectant for O. seepicola and M. seepicolus when grown at 4°C, the *in situ* cold seep temperature. The metagenomic and experimental data led to the proposal of a model (Fig. 8) for how the cold seep bacteria and methanogens syntrophically convert GBT to methane, which ultimately contributes to the sourcing of biogenic methane in cold seeps.

GBT is an osmoprotectant used by a wide variety of organisms, particularly those that live in oceans. Consequently, GBT is ubiquitous in coastal and marine sediments. GBT can be degraded through oxidative or reductive pathways. In oxic environments, it is demethylated by monooxygenases to yield dimethylglycine and/or sarcosine monomethylglycine and glycine (10). In anoxic environments, GBT is either reduced to TMA and acetyl phosphate or demethylated to dimethylglycine (23, 33, 34) with a methyl group being transferred to a specific methyl carrier. The selenocysteine-containing GBT reductase complex (GrdHI) is key to GBT reduction, while thioredoxin (TrxA) and its reductase (TrxB) transfer electrons from NAD(P)H to GBT reductase. In addition, *grdABCD* encodes a reductase complex that catalyzes glycine, sarcosine, or GBT reduction. A BCCT-type GBT transporter, OpuD, imports GBT to cells. The anaerobic GBT demethylation can also be accomplished by methanogenic archaea (23), sulfate-reducing bacteria (34), and acetogenic bacteria (35) using GBT transferase (MtgB). These anaerobes typically further metabolize the methyl group generated by GBT demethylation, and it is either transferred to coenzyme M by methanogens in methane production or through the Wood-Ljungdahl pathway (WLP) to produce acetate in bacteria (36, 37).

The genes involved in the two pathways of anaerobic GBT metabolism were all identified in metagenomic sequencing contigs derived from the Formosa cold seep sediment communities. Most of the genes involved in GBT reduction were derived from *Clostridium* and a cold seep bacterium *Psychrilyobacter* affiliated with the *Fusobacteriaceae* family. In contrast, the identified MtgB homologs were likely from additional species, mainly *Firmicutes* and *Alphaproteobacteria* (24). Similarly, genes identified that could be involved in GBT demethylation were attributed to *Chloroflexi*, *Clostridium*, and *Alphaproteobacteria*. Consequently, the two GBT metabolism pathways were simultaneously active in this cold seep.

The synergism of bacteria, particularly sulfate-reducing bacteria and methanogenic archaea in the conversion of GBT to methane, has been observed in marine ecosystems and coastal saltmarsh sediments (26, 30). Although different bacterial species implicated in GBT reduction were observed in the Formosa cold seep, the syntrophic methanogens all belonged to the *Methanococcoides* genus. Nevertheless, *Methanococcoides* strains from diverse marine habitats only demethylate some GBT (23), while M. seepicolus is capable of completely converting GBT to methane (Fig. 5b) and, presumably, dimethylglycine (DMG). A novel GBT-utilizing *Methanolobus* strain was isolated from an estuary (21), which, together with M. seepicolus, increases the known diversity of methanogens that are capable of directly using GBT. Furthermore, the *Methanococcoides* strains that exhibit high 16S rRNA gene identities with M. seepicolus are widely distributed in subseafloor sediments (Table S4), indicating that GBT demethylation mediated by methanogenic archaea could be a prevalent pathway in marine ecosystems.

10.1128/mSystems.00703-21.7 TABLE S4 Distribution of *Methanococcoides* showing high 16S rRNA sequence identity with M. seepicolus strain. Download Table S4, DOCX file, 0.03 MB. Copyright © 2021 Li et al. 2021 Li et al. https://creativecommons.org/licenses/by/4.0/ This content is distributed under the terms of the Creative Commons Attribution 4.0 International license.

A 1:2.6 ratio of GBT to methane was observed in the consortium of O. seepicola and M. seepicolus, which differs from the 1:1 molecular ratio of GBT degradation to methane production previously observed in an estuarine microcosm (23). However, the ratio of 0.87 (2.6/3) methane from each methyl group is higher than the theoretical 0.75 mol methane per methyl group that is predicted from the canonical methylotrophic methanogenic pathway. Given that decreasing GBT degradation rates coincided with decreases in TMA accumulation and methane production initiation in the coculture, we assume that some of the reducing equivalent generated during GBT reduction by O. seepicola could be used during TMA-derived methane production. The co-occurrence of GBT degradation with TMA degradation has also been observed in estuarine microcosms (26).

Both GBT and DMG have been documented as compatible solutes in halotolerant and halophilic methanogenic archaea (38). DMG has also been observed to exhibit better heat and cold stress-relieving properties in Bacillus subtilis, which uses the ABC transporter OpuA to take up DMG (32). Here, evidence is provided that DMG, and presumably GBT, functions as a cryoprotectant for cold seep microorganisms, while MtgB, which is involved in DMG formation from GBT demethylation, was also encoded in the genomes of diverse cold seep bacterial species (Fig. S1b).

In conclusion, this work reports that the osmotic stress protectant, GBT, contributes to most of the biogenic methane produced in an SCS cold seep. In addition, a new bacterial species, O. seepicola, affiliated with the *Clostridiaceae* family, and a new methanogen species, M. seepicolus, which were both isolated from the cold seep, could synergistically convert GBT to methane. Moreover, M. seepicolus also demethylates GBT to methane and DMG, a cryoprotectant that promoted the growth of cold seep organisms at *in situ* low temperatures. GBT is an osmoprotectant ubiquitously used in marine organisms, including the cold seep benthic community, and the *grdH* genes are also found in the Gulf of Mexico cold seep. Thus, the GBT-dependent biogenic methane production pathway identified here could be distributed among global cold seeps.

## MATERIALS AND METHODS

### Cold seep sediment sampling.

Samples were collected from the Formosa cold seep of SCS (Fig. 1a) during the research vessel “KEXUE” scientific cruises in 2019. Reduced black sediments were collected with a gravity corer. Onboard, the top layer that is affected by seawater was removed, and the sediment column was sectioned every 20 cm, with the sediment from each section center stored in anaerobic bio-bags at 80°C until further use.

### Development of methane-producing microcosms of cold seep sediments using various methanogenic precursors.

Approximately 0.5 g of cold seep sediment was inoculated into 5 ml of prereduced artificial seawater containing a final concentration of 20 mM TMA, GBT, acetate, choline, or 80% H_2_-20% CO_2_ gas to develop methane-producing microcosms. Each microcosm was placed in a 15-ml anaerobic tube and placed under 10^5^ Pa of headspace gas of N_2_, except for the H_2_-CO_2_ treatment. A microcosm without methanogenic precursors was included as a control, and another microcosm without methanogenic precursor was amended with ampicillin and kanamycin, each at a final concentration of 100 mM to suppress bacterial growth. All the enrichments were incubated at 18°C, and methane production was monitored using gas chromatography as described below. Each experiment was conducted with four replicates.

### Isolation, identification, and characterization of GBT-degrading bacteria and methanogenic archaea.

Bacterial and methanogenic archaeal strains were isolated using GBT as the sole carbon source from the GBT microcosm using the Hungate rolling tube method (39). About 0.5 ml of microcosm slurry was used as the inoculant and then diluted over a 10-fold series into 5 ml prereduced YTG (40) agar medium containing 20 mM GBT. Ampicillin and kanamycin, each at a 100 mM final concentration, were added to promote isolation of methanogenic archaea. The Hungate roll tubes were incubated at 18°C, and single colonies were picked inside an anaerobic glove box (Thermo Fisher, USA).

Cell morphologies were examined with an optical microscope (BX40; Olympus). Substrate utilization by strains ZWT and LLY was assayed in YTG basal medium by omitting yeast extract, tryptone, and glucose, and with 20 mM of each test substance individually.

Growth of the bacterial strain ZWT and the methanogenic strain LLY were determined by optical density at 600 nm (OD_600_) measurements and methane production, respectively. Growth characteristics were assayed in YTG basal medium containing GBT. Growth temperatures were evaluated at 0, 4, 8, 18, 22, 30, and 37°C and pH-dependent growth at pH 3.0, 4.0, 5.0, 6.0, 6.5, 7.0, 7.5, 8.0, 8.5, 9.0, and 10.0. NaCl tolerance was determined at 0.0, 0.5, 1.0, 1.5, 2.0, 2.5, 3.0, 3.5, 4.0, 4.5, 5.0, 6.0, 7.0, and 7.5%. All the tests were performed in triplicate.

### Measurements of methane, glycine betaine, and trimethylamine and carbon isotope analysis of CH_4_ and CO_2_.

Methane was measured using a GC-14B gas chromatograph (GC; Shimadzu, Japan) equipped with a flame ionization detector and a C_18_ column, as previously described (41). GC temperature parameters included the column at 50°C, the injector at 80°C, and the detector at 130°C.

TMA concentration in cultures was quantified using an Agilent Technologies 6890N GC-5973N MSD equipped with a DB-Wax column (60 m × 0.25 mm [inside diameter], film thickness of 0.15 μm; J&W Scientific, Folsom, CA). Nitrogen was used as the carrier gas at a constant flow rate of 1 ml/min. The temperature of the injector was set at 280°C, the gas chromatography-mass spectrometry (GC-MS) transfer line at 280°C, the ion source at 230°C, and the quadrupole at 150°C. The reference standard solution contained 0.25 to 40 mM TMA. Spent culture (0.5 μl) was filtered through a 0.22-μm Millex GP filter (Millipore Express; polyethersulfone [PES] membrane) prior to assay measurements.

GBT was quantified using a Shim-pack GIS hydrophilic interaction liquid chromatography (HILIC) column (4.6 mm by 250 mm, 5 μm; ShimSen) that was eluted with acetonitrile/water (85:15) at a flow rate of 1.0 ml·min^−1^ at 30°C. Spent culture liquid (200 μl) was purified using an MCX column (60 mg/3 ml; ShimSen), filtered with a 0.22-μm nylon centrifuge tube filter (Costar, Corning, NY, USA), and analyzed with a UV detector at a 195-nm wavelength.

The carbon isotope signatures of methane and CO_2_ were determined using an isotope ratio mass spectrometry coupled with gas chromatography (GC-IRMS)instrument (Thermo Fisher Scientific, Germany) (42), with ^13^C/^12^C isotope ratios expressed as δ^13^C values (%) relative to the Vienna Pee Dee Belemnite (V-PDB) standard.

### Illumina sequencing of archaeal and bacterial 16S rRNA genes.

The genomic DNAs of strains ZWT and LLY were extracted using the FastDNA Spin kit for soil (MP BioScience, Derby, UK) and quantified using a NanoDrop 2000 spectrophotometer (Thermo Fisher). The 16S rRNA genes of the cultures were amplified by PCR using the universal bacterial 16S rRNA gene amplification primers 27F (5′-AGAGTTTGATCCTGGCTCAG-3′) and 1492R (5′-GGTTACCTTGTTACGACTT-3′) and the universal methanogen 16S rRNA primers Met86F (5′-GCTCAGTAACACGTGG-3′) and Met1340R (5′-CGGTGTGTGCAAGGAG-3′), both with corresponding PCR annealing temperatures of 55°C. PCR products were ligated into T-vector plasmids, and the 16S rRNA genes were sequenced at Biosune Biotechnology Co., Ltd. (Shanghai, China).

Total DNA was extracted from the *in situ* sediments (∼0.5 g) and GBT-enriched microcosms (∼0.5 ml) using a MoBio PowerSoil DNA isolation kit (MoBio, Carlsbad, CA, United States) according to the manufacturer’s protocol. The primer pairs 519F and 915R and 341F and 806R were used to amplify a 399-bp fragment of the V4-V5 hypervariable regions of archaeal 16S rRNA genes (43) and a 456-bp fragment of bacterial 16S rRNA genes (44), respectively. The purified amplicons were sequenced on the Illumina MiSeq platform at Novogene (Beijing, China) with standard protocols. Sequencing data were processed and analyzed using the QIIME (version 1.7.0) pipeline (45).

### Genomic and metagenomic analysis.

Genomes and microcosm community DNAs were paired-end sequenced on the Illumina HiSeq 2000 platform at Novogene (Beijing, China), followed by processing of data with standard protocols. The raw paired-end metagenomic sequencing reads of two replicate samples were first quality filtered and quality controlled using the read_qc module of metaWRAP (46). Clean reads generated for each sample were individually assembled into contigs using the MEGAHIT v1.1.3 assembler (47) with default parameters, followed by removal of short contigs (<1,000 bp). Nonredundant gene catalogs were generated as previously described (48). Briefly, open reading frames (ORFs) were predicted using MetaGeneMark (49), and those of length >100 bp were retained. Redundant ORFs and contigs were removed using CD-HIT (v4.7) (50). Clean reads were then mapped to ORFs or contigs and enumerated with CoverM (https://github.com/wwood/CoverM), followed by calculation of relative abundances of genes/contigs in each sample based on the proportion of mapped reads divided by the gene/contig length. Taxonomic assignments of each ORF were generated by mapping the amino acid sequences against the NCBI nr database (https://www.ncbi.nlm.nih.gov/) using Diamond (51). Functional annotations for ORFs were predicted with eggNOG 5.0 (52) and KofamKOALA (53), and the hit score above predefined threshold of each knockout (KO) was selected.

### Quantitative reverse transcription-PCR.

Total RNA was extracted from mono- and cocultures using the RNAiso Plus kit (TaKaRa, Dalian, China) according to the manufacturer’s instructions. RNA extracts were then treated with RNase-Free DNase (TaKaRa) to remove contaminating DNA, and cDNA was synthesized using the Moloney murine leukemia virus (M-MLV) reverse transcriptase cDNA synthesis kit (TaKaRa) following the manufacturer’s instructions. The transcript copies of *grdH*, *mtgB*, *oupD*, and *mttB* were quantified via qPCR using the respective primers listed in Table S5. To estimate mRNA copies of the evaluated genes, a standard curve was generated for each gene by qPCR using 10-fold serially diluted PCR products as the templates. We used 16S rRNA gene copy numbers of corresponding strains as proxies for biomass. All the measurements were performed on triplicate samples and repeated at least three times. qPCRs were performed using an SYBR Premix Ex Taq kit (TaKaRa Bio Inc., Japan) in triplicate experiments and were conducted in an ABI Prism 7000 detection system (Applied Biosystems USA). Each qPCR mixture contained 12.5 μl of SYBR qPCR mix (Toyobo), 5 μl of cDNA, 100 nM each primer, and double-distilled H_2_O to a final volume of 25 μl. PCRs were initiated at 95°C for denaturation over 30 s, followed by 35 cycles of amplification comprising 95°C at 10 s, 55°C at 30 s, and 72°C at 30 s. Fluorescence data were collected during the elongation stages.

10.1128/mSystems.00703-21.8 TABLE S5 RT-PCR primers used in this study. Download Table S5, DOCX file, 0.02 MB. Copyright © 2021 Li et al. 2021 Li et al. https://creativecommons.org/licenses/by/4.0/ This content is distributed under the terms of the Creative Commons Attribution 4.0 International license.

### Phylogenetic analyses.

The 16S rRNA gene sequences were retrieved from the genomes of strains ZWT and LLY, and related 16S rRNA gene references were retrieved from the SILVA database for phylogenetic analysis. Phylogenetic trees were constructed using neighbor-joining methods in the MEGA 6.0 software program. Node support values were determined using 1,000 replicate bootstraps.

GrdH and MtgB phylogenies were generated from proteins encoded in metagenomic contigs for the *in situ* cold seep sediments and the GBT-enriched microcosms, along with references retrieved from the NCBI database. The amino acid sequences were aligned with the MUSCLE aligner, and neighbor-joining trees were constructed using the MegaX program. Node support for both trees was evaluated by 1,000 bootstrap replicates. The resulting trees were edited and visualized with iTOL (54).

### Data availability.

All of the data supporting the findings of this study are available in the paper and the supplemental material. The Illumina sequencing data of archaeal and bacterial 16S rRNA gene V4-V5 hypervariable regions are deposited in the NCBI Sequence Read Archive under accession numbers SRR14320062 to SRR14320068. The 16S rRNA gene sequences for amplicons from strains ZWT and LLY are deposited in NCBI GenBank under accession numbers MW674679 and MW675309, respectively. The raw metagenome sequencing data were deposited under the accession numbers SRR14270296, SRR14270297, and SRR14339844 to SRR14339846 in the NCBI Sequence Read Archive. The metagenomic contig data for the sediment and GBT enrichment are available through figshare at https://doi.org/10.6084/m9.figshare.14447793.v1.
