# Supplementary figures and images for: Bacteria and Archaea Synergistically Convert Glycine Betaine to Biogenic Methane in the Formosa Cold Seep of the South China Sea
Source: mSystems. 2021 Sep 7;6(5):e00703-21. doi: 10.1128/mSystems.00703-21 (PMC8547467; doi:10.1128/mSystems.00703-21)

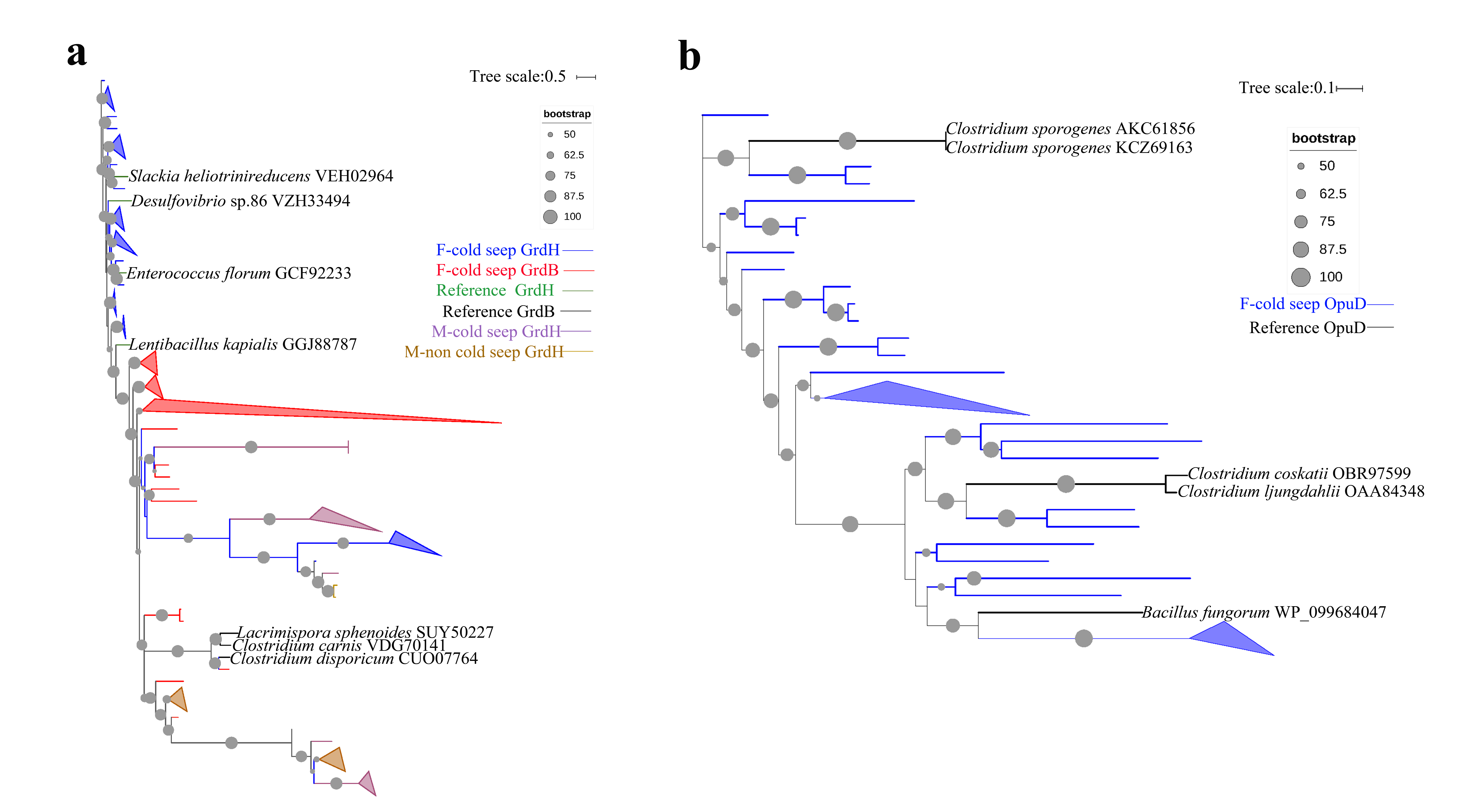

Supplement: FIG S1 [file msystems.00703-21-sf001.tif]

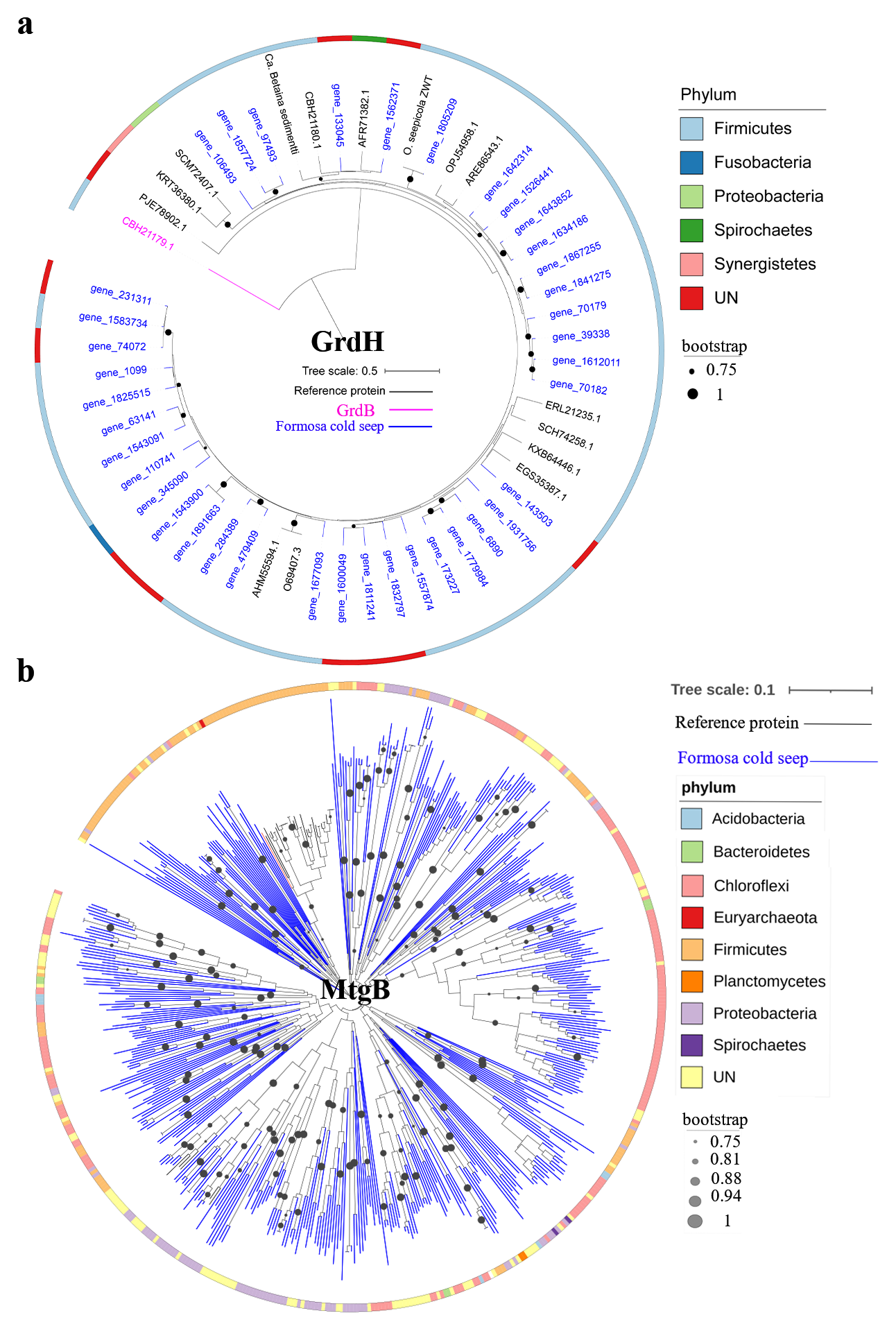

Supplement: FIG S2 [file msystems.00703-21-sf002.tif]

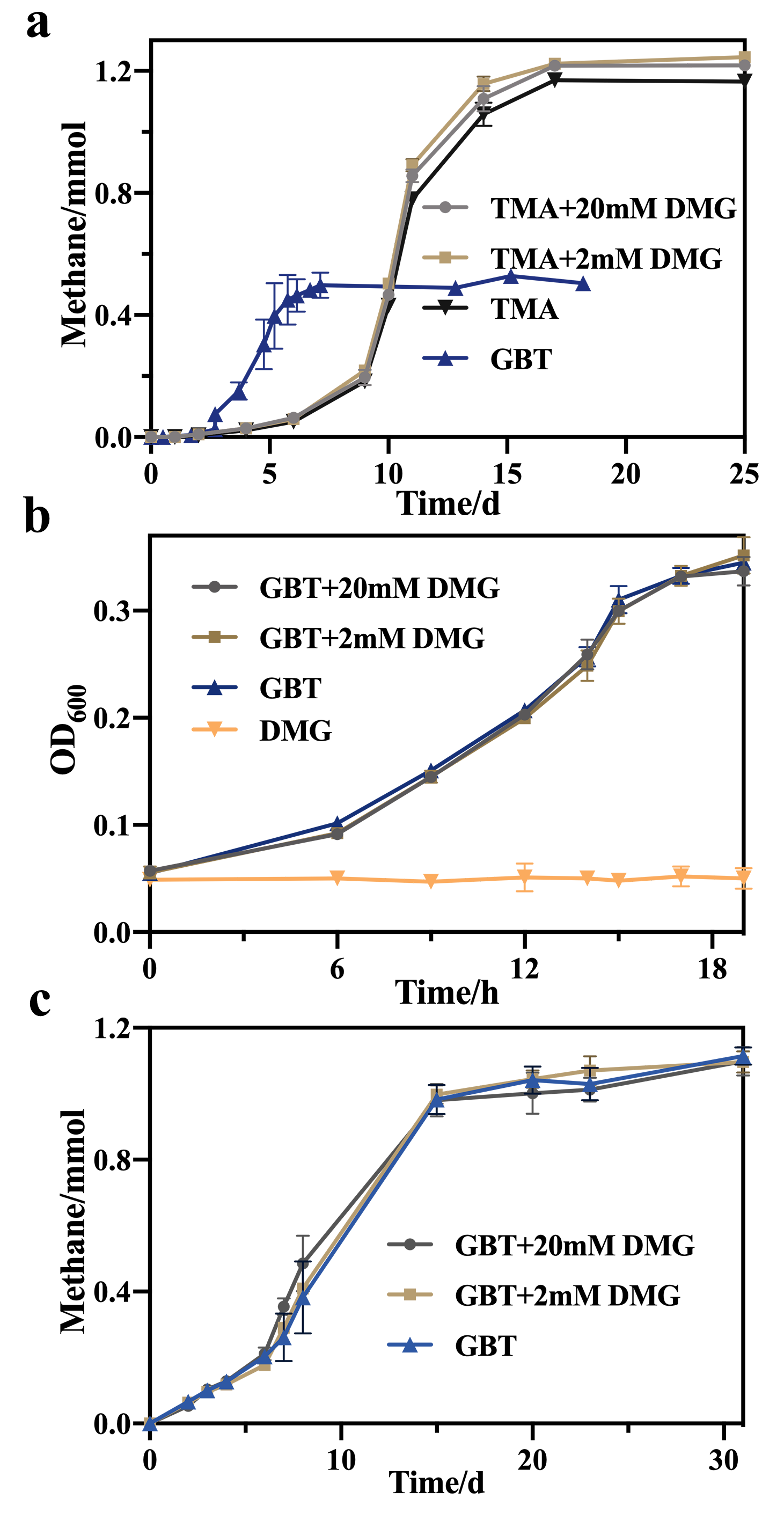

Supplement: FIG S3 [file msystems.00703-21-sf003.tif]
