# Supplementary material for: Bacteria and Archaea Synergistically Convert Glycine Betaine to Biogenic Methane in the Formosa Cold Seep of the South China Sea
Source: mSystems. 2021 Sep 7;6(5):e00703-21. doi: 10.1128/mSystems.00703-21 (PMC8547467; doi:10.1128/mSystems.00703-21)
Supplement: TABLE S1 [file msystems.00703-21-st001.docx]

**Table S1. Differential phenotypic and physiological characteristics of strain ZWT and its closest phylogenetic relative**

| Feature | *Oceanirhabdus seepicola*  ZWT | | *Oceanirhabdus sediminicola*  NH-JN4 |
| --- | --- | --- | --- |
| Isolation source | Cold seep sediment | Sea sediment | |
| Cell width (µm) | 0.5 – 1.3 | 0.5 – 1.2 | |
| Cell length (µm) | 3.0 – 4.5 | 2.2 – 7.0 | |
| Temperature (℃) |  |  | |
| Optimum | 18 – 22 | 34 – 38 | |
| Range | 4 – 30 | 22 – 42 | |
| pH |  |  | |
| Optimum | 7.0 – 8.5 | 6.5 – 7.0 | |
| Range | 4.0 – 10.0 | 6.0 – 8.5 | |
| NaCl concentration (%) |  |  | |
| Optimum | 3.0 – 4.5 | 2.5 | |
| Range | 1.5 – 8 | 0.5 – 6 | |
| Carbohydrate fermentation |  |  | |
| Glucose | + | - | |
| Maltose | + | - | |
| Amino acid fermentation |  |  | |
| Glycine  Glycine betaine  Choline | +  +  - | +  - | |
| %16S rRNA gene sequence similarity to strain NH-JN4 | 94.12 | 100 | |
