# Supplementary material for: Bacteria and Archaea Synergistically Convert Glycine Betaine to Biogenic Methane in the Formosa Cold Seep of the South China Sea
Source: mSystems. 2021 Sep 7;6(5):e00703-21. doi: 10.1128/mSystems.00703-21 (PMC8547467; doi:10.1128/mSystems.00703-21)
Supplement: TABLE S2 [file msystems.00703-21-st002.docx]

**Table S2. Genome statistics**

| Feature | *Oceanirhabdus seepicola* ZWT | *Methanococcoides seepicolus* LLY |
| --- | --- | --- |
| Genome size（bp） | 5335032 | 2803900 |
| DNA G + C content (mol %) | 31.05 | 41.73 |
| % DNA coding region | 86.06 | 82.36 |
| % Genome(internal) | 13.94 | 17.64 |
| Genes mean G + C content (mol %) | 26.53 | 43.25 |
| rRNA genes (5S-16S-23S) | 1-1-1 | 6-1-1 |
| tRNA genes | 72 | 40 |
| Protein coding genes | 5017 | 2800 |
| Median size of protein coding genes (bp) | 915 | 825 |
| Gene products assigned to arCOGs | 2751 | 1833 |
| Gene products assigned Pfam domains | 3433 | 1805 |
| CRISPR repeats | 2 | 4 |
| Glycine betaine metabolic genes | *grdIH-opuD** | *opuD-mtgBC** |

**grdH*, GBT reductase complex component B subunit beta; *grdI*, GBT reductase complex component B subunit alpha;

*opuD*, GBT transporter; *mtgB*, GBT corrinoid protein: CoM-methyltransferase; *mtgC*, GBT corrinoid protein.
