# Supplementary material for: Bacteria and Archaea Synergistically Convert Glycine Betaine to Biogenic Methane in the Formosa Cold Seep of the South China Sea
Source: mSystems. 2021 Sep 7;6(5):e00703-21. doi: 10.1128/mSystems.00703-21 (PMC8547467; doi:10.1128/mSystems.00703-21)
Supplement: TABLE S3 [file msystems.00703-21-st003.docx]

**Table S3. Differential phenotypic and physiological characteristics of strain LLY and its closest phylogenetic relatives**

|  | *Methanococcoides seepicolus*  LLY | *Methanococcoides burtonii*  DSM 6242^T^ | *Methanococcoides alaskense*  AK-5^T^ |
| --- | --- | --- | --- |
| DNA G+C content (mol%) | 41.73 | 39.6 | 41.9 |
| Extracellular structures | Flagella | Flagella | Pili |
| Motility | NR | + | - |
| Range for growth |  |  |  |
| Temperature (℃) | 0 – 25 | 1.7 – 29.5 | -2.3 – 30.6 |
| Optimum temperature (℃) | 18 | 23.4 | 23.6 |
| Na^+^ (M) | 0.25 – 0.5 | 0.2 – 0.5 | 0.1 – 0.7 |
| pH | 7.5 – 9.0 | 6.8 – 8.2 | 6.3 – 7.5 |
| Catabolic substrates: |  |  |  |
| Methanol | + | + | - |
| Glycine betaine | + | - | - |
