# Supplementary material for: Bacteria and Archaea Synergistically Convert Glycine Betaine to Biogenic Methane in the Formosa Cold Seep of the South China Sea
Source: mSystems. 2021 Sep 7;6(5):e00703-21. doi: 10.1128/mSystems.00703-21 (PMC8547467; doi:10.1128/mSystems.00703-21)
Supplement: TABLE S4 [file msystems.00703-21-st004.docx]

**Table S4. Distribution of *Methanococcoides* showing high 16S rRNA sequence identity with strain *M.seepicolus***

| **Accession** | **Definition** | **Identity (%)** | **Environmental sample** | **Reference** |
| --- | --- | --- | --- | --- |
| DQ280485 | Methanomicrobiales archaeon | 99.86 | Skan Bay, Alaska | 1 |
| MK568483 | *Methanococcoides* sp. strain 62 | 99.58 | Guaymas basin | Direct submission to NCBI |
| LC183843 | Uncultured archaeon | 99.70 | Atumi No.2 Knoll, Japan | Direct submission |
| AB598271 | *Methanococcoides* sp. MO-MCD | 99.79 | Shimokita Peninsula, Japan | 2 |
| NR_118979 | *Methanococcoides burtonii* | 99.18 | Ace Lake, Antarctica | Direct submission to NCBI |
| AY941802 | *Methanococcoides alaskense* strain AK-9 | 99.79 | Skan Bay, Alaska | 3 |
| NR_029122 | *Methanococcoides alaskense* strain AK-5 | 99.78 | Skan Bay, Alaska | 3 |
| JN123668 | Uncultured archaeon | 99.01 | Tainan Ridge, Taiwan | Direct submission to NCBI |
| GU553549 | Uncultured archaeon | 98.86 | Yung-An Ridge, Taiwan | Direct submission to NCBI |
| AF354132 | Uncultured archaeon | 99.24 | methane seep, Californian | Direct submission to NCBI |

**References**

1. Kendall, M.M., Wardlaw, G.D., Tang, C.F., Bonin, A.S., Liu, Y. and Valentine, D.L. 2007. Diversity of Archaea in marine sediments from Skan Bay, Alaska, including cultivated methanogens, and description of *Methanogenium boonei* sp. nov. Appl Environ Microbiol 73:407-414.

2. Imachi, H., Aoi, K., Tasumi, E., Saito, Y., Yamanaka, Y., Saito, Y., Yamaguchi, T., Tomaru, H., Takeuchi, R., Morono, Y. et al. 2011. Cultivation of methanogenic community from subseafloor sediments using a continuous-flow bioreactor. ISME J 5:1913-1925.

3. Singh, N., Kendall, M.M., Liu, Y. and Boone, D.R. 2005. Isolation and characterization of methylotrophic methanogens from anoxic marine sediments in Skan Bay, Alaska: description of *Methanococcoides alaskense* sp. nov., and emended description of *Methanosarcina baltica*. Int J Syst Evol Microbiol 55:2531-2538.
