# Supplementary material for: Bacteria and Archaea Synergistically Convert Glycine Betaine to Biogenic Methane in the Formosa Cold Seep of the South China Sea
Source: mSystems. 2021 Sep 7;6(5):e00703-21. doi: 10.1128/mSystems.00703-21 (PMC8547467; doi:10.1128/mSystems.00703-21)
Supplement: TABLE S5 [file msystems.00703-21-st005.docx]

**Table S5. RT-PCR primers used in this study**

| Strain Gene Primers Sequence（5’-3’） | | | | |  |
| --- | --- | --- | --- | --- | --- |
| *O. seepicola*  ZWT  *M. seepicolus*  LLY | *16S rRNA* | Och16S-F | TCTCCCGCACTCTAGATAAC |  |  |
|  |  | Och16S-R | AAAGCCCTGTCTTCGAGGA |  |  |
|  | *grdH*  *opuD* | 3076F | AGAGCAGGAAGATACGGAAGTG |  |  |
|  |  | 3076R | ACCAGCTGCACTCTTAGCTC |  |  |
|  |  | 3078F | CACTGGGGATTAGCTCCTTGG |  |  |
|  |  | 3078R | GTTGCCATACCAGCAACTGT |  |  |
|  | *16S rRNA* | Mcc16S-F | ACCCATCATCCCGAAGGAC |  |  |
|  |  | Mcc16S-R | CGGGTGGAGCCTGCGGTTTA |  |  |
|  | *opuD* | 2058F | ATTCGTTGGTCAGTTCATTGC |  |  |
|  |  | 2058R | TCAAGTCCGAGTGCGTTAC |  |  |
|  | *mtgB* | 2059F | AGTAATGGCGGGTTCGACAG |  |  |
|  |  | 2059R | AGGATTTACGCACTGTGCCA |  |  |
|  | *mttB* | 0619F | CCGTGACAAGAAG | | |
|  |  | 0619R | TCCCTTGCGGAAAC | | |
